# Supplementary material for: A palisade-shaped membrane reservoir is required for rapid ring cell inflation in Drechslerella dactyloides
Source: Nat Commun. 2023 Nov 15;14:7376. doi: 10.1038/s41467-023-43235-w (PMC10651832; doi:10.1038/s41467-023-43235-w)

A palisade-shaped membrane reservoir is required for rapid ring cell inflation in *Drechslerella dactyloides*

Yue Chen[1], Jia Liu[1], Seogchan Kang[2], Dongsheng Wei[1], Yani Fan[3,4]*, Meichun Xiang[3,4]*, and Xingzhong Liu[1,3]*

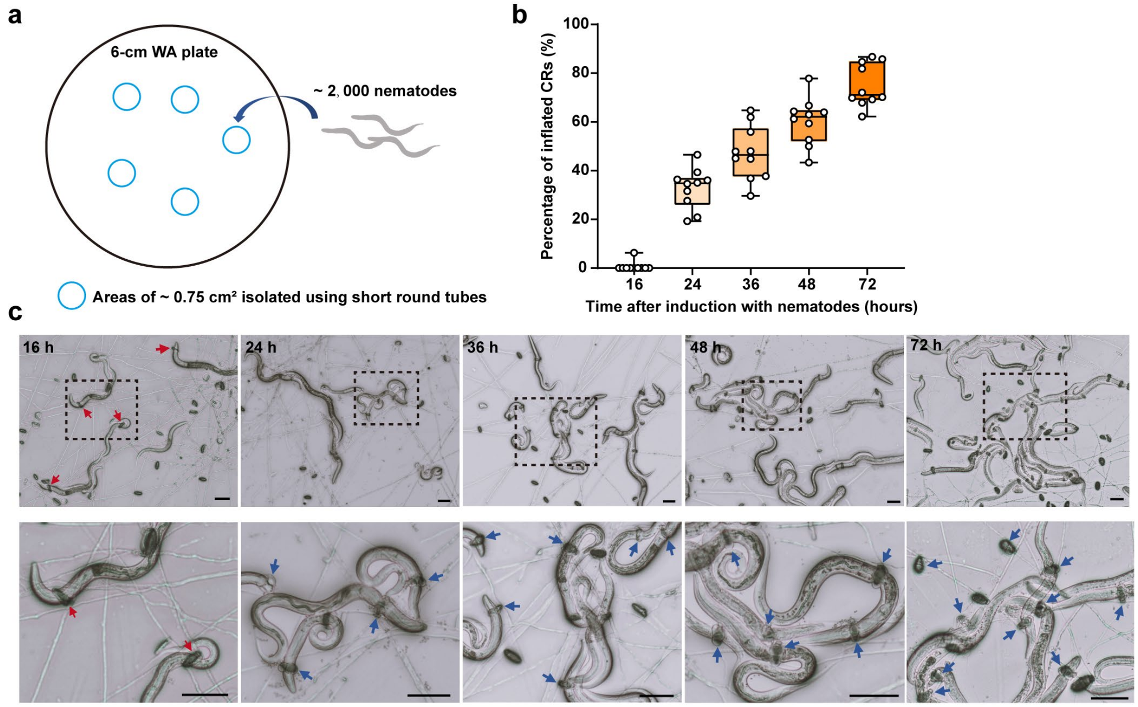

Supplementary Fig. 1. Measurement of CR inflation using nematodes. a A diagram illustrating how we measured CR inflation by applying ~2,000 nematodes in each area (noted by blue circles) to ensure nematodes entry through all CRs in these areas. **b** Boxplot showing the percentages of inflated CRs after applying nematodes (n = 10 microscopic fields of view from 2 biological replicates). The middle line in the boxplot displays the median, edges represent the upper and lower quartiles, and whiskers indicate minimum and maximum values. Source data are provided as a Source Data file. **c** Light micrographs showing CR inflation after applying nematodes. The lower panel shows magnified views of the areas noted by the dotted black box in the upper panel. Red and blue arrows denote uninflated and inflated CRs, respectively. Scale bars = 50 µm. Data shown is a representative from three independent experiments.

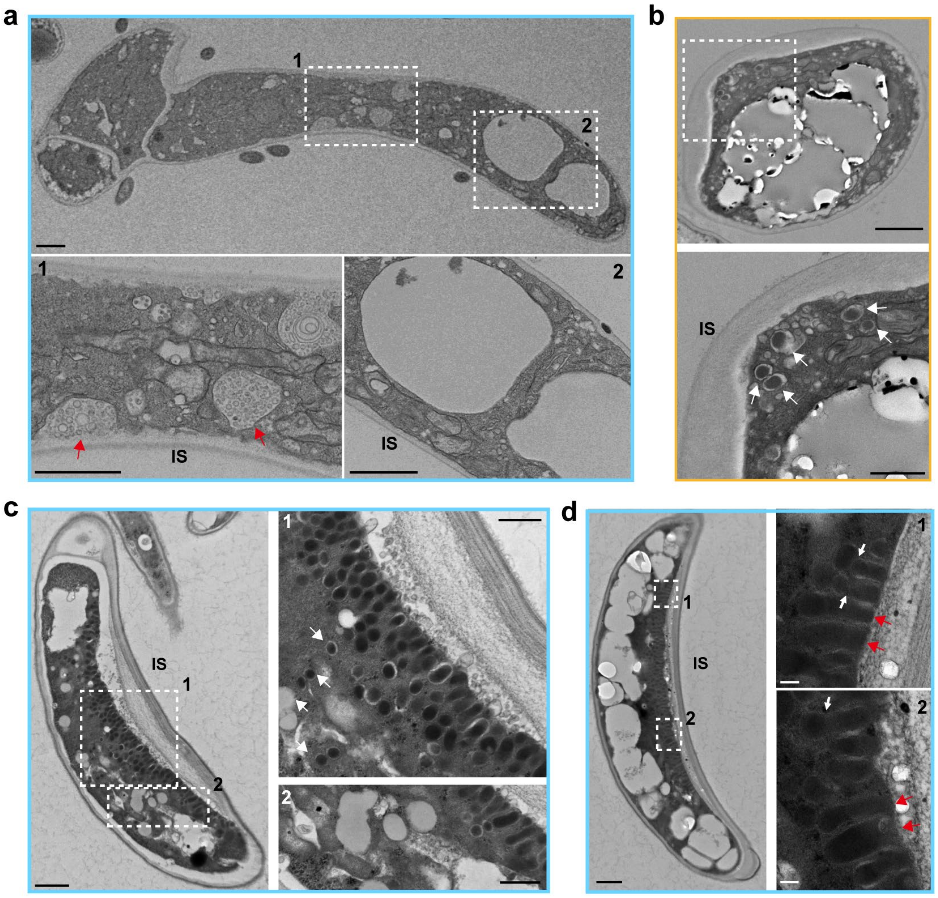

Supplementary Fig. 2. Transmission electron micrographs of the ring cells at different stages of CR formation. a Images of an immature CR. Red arrows indicate the exosome like structures. IS, inner side of ring cell. Scale bars = 1 µm. **b, c** Images of immature CRs show vesicles (noted by white arrows) accumulating at the inner side (IS) of ring cells. In b, scale bars (upper panel = 1 µm; lower panel = 0.5 µm); In c, scale bars (left panel = 1 µm; right panel = 0.5 µm). **d** Images of PMS formed at the inner side (IS) of ring cells. Strings of fused vesicles connected to the plasma membrane (PM) were indicated using red arrows. White arrows indicate the fusion point between vesicles. Scale bars (left panel = 1 µm; right panel = 0.2 µm). Magnified views of the areas denoted by white dotted box in **a-b** and **c-d** are shown at the bottom and right side, respectively. The blue and yellow borders denote the transverse and longitudinal sections, respectively. The images shown were chosen among those collected from at least 5 different CRs.

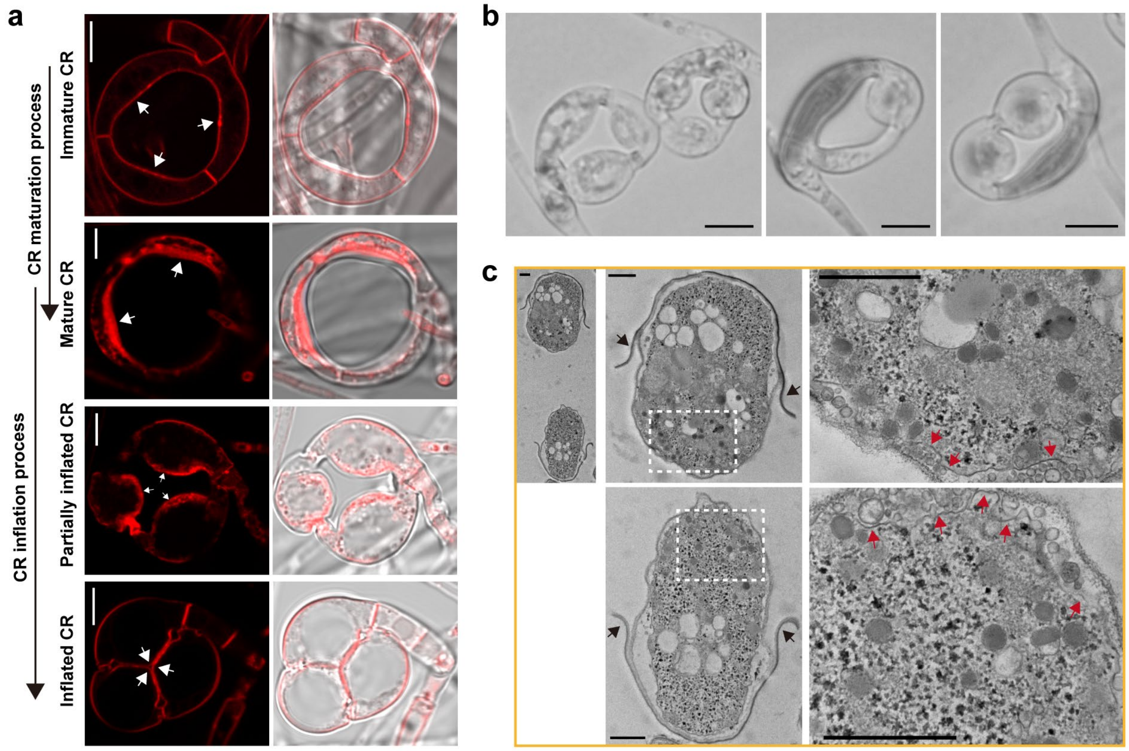

Supplementary Fig. 3. Features of the ring cells at different stages. a The PM of CRs at different stages was stained using FM4-64. White arrows indicate the PM at the inner side of ring cells. Scale bars = 6 µm. **b** Light micrographs showing partially inflated CRs. Scale bars = 10 µm. In **a** and **b**, representative images from three independent experiments are shown. **c** Transmission electron micrographs of two partially inflated ring cells. Black arrows denote the ruptured outer cell wall. Red arrows indicate the wavy and curved PM. Scale bars = 1 µm. Magnified views of the areas denoted by white dotted box are shown on the right side. The images shown were chosen among those collected from at least 5 different CRs.

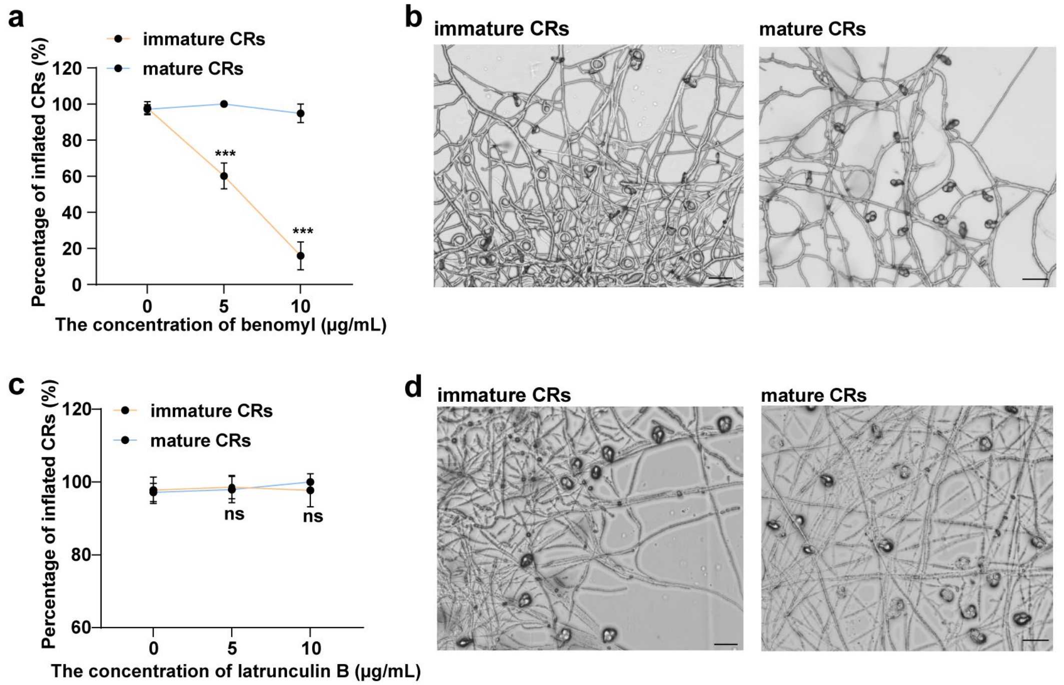

Supplementary Fig. 4. Effect of cytoskeleton polymerization inhibitors on CR maturation and inflation. a, c The percentages of inflated CRs after exposing immature and mature CRs to 5 µg/mL and 10 µg/mL benomyl (**a**) and 5 µg/mL and 10 µg/mL latrunculin B (**c**) for 24 hours. n = 5 biological replicates (**a**), 3 biological replicates (**c**). Two-tailed t-test, mean ± SD. In **a**, ***$P = 5.94 \times 10^{-6}$ (5 µg/mL), 2.38×10^{-8} (10 µg/mL). ns, not significant. Source data are provided as a Source Data file. **b, d** Light micrographs showing CR inflation after exposing immature and mature CRs to 10 µg/mL benomyl (**b**) and 10 µg/mL latrunculin B (**d**) for 24 hours. Scale bars = 50 µm. Data shown is a representative from three independent experiments.

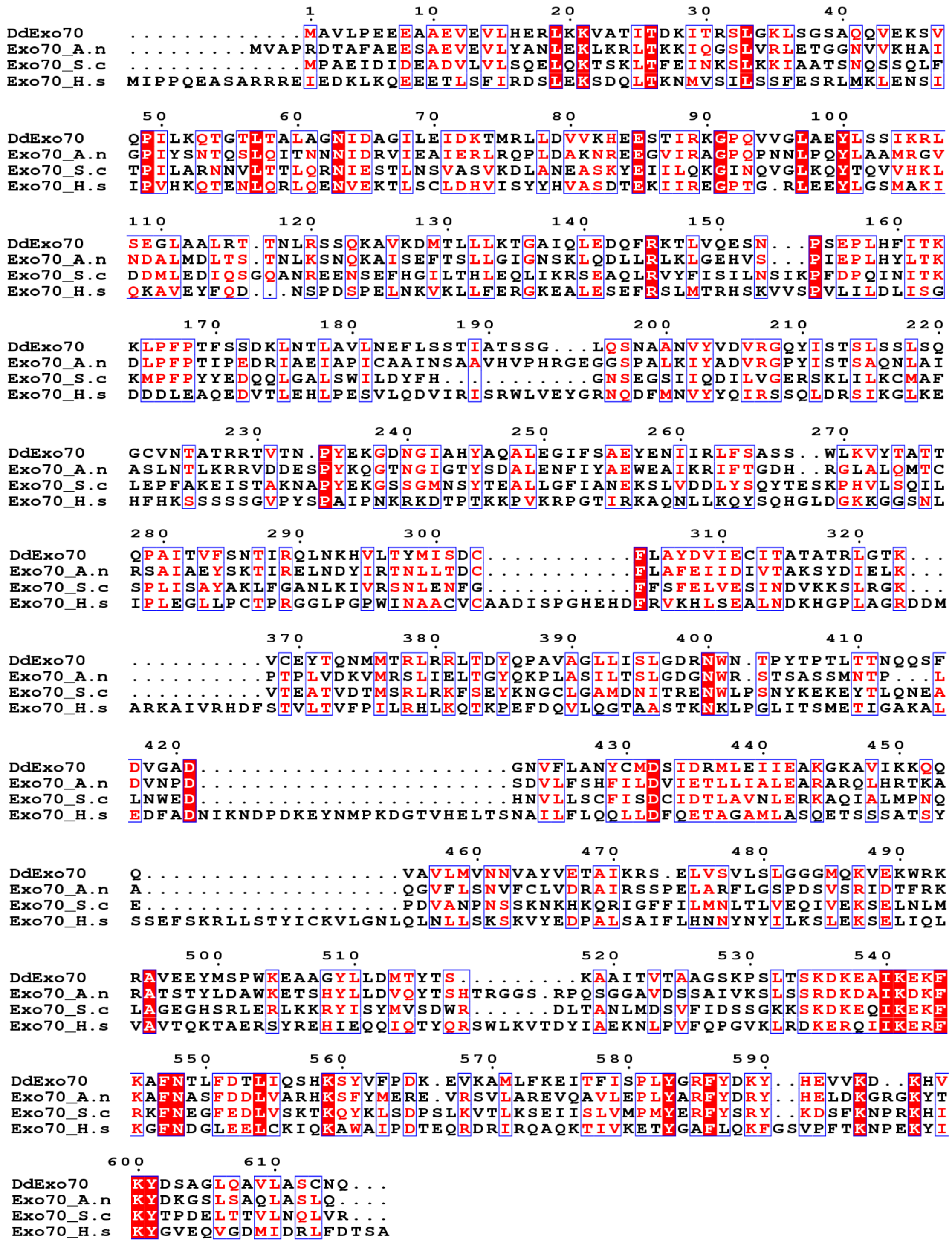

Supplementary Fig. 5. Sequence alignment of four Exo70 proteins. Sequences of the Exo70 proteins encoded by *D. dactyloide*, *S. cerevisiae* (NP_012450, https://www.ncbi.nlm.nih.gov/protein/NP_012450.1/), *A. nidulans* (XP_663814, https://www.ncbi.nlm.nih.gov/protein/XP_663814), and *Homo sapiens* (NP_001013861.1,

https://www.ncbi.nlm.nih.gov/protein/NP_001013861.1) were aligned. Similar amino acids are indicated by red text, and identical amino acids are noted using the red background. The amino acid sequences of DdExo70 is provided in Source Data file.

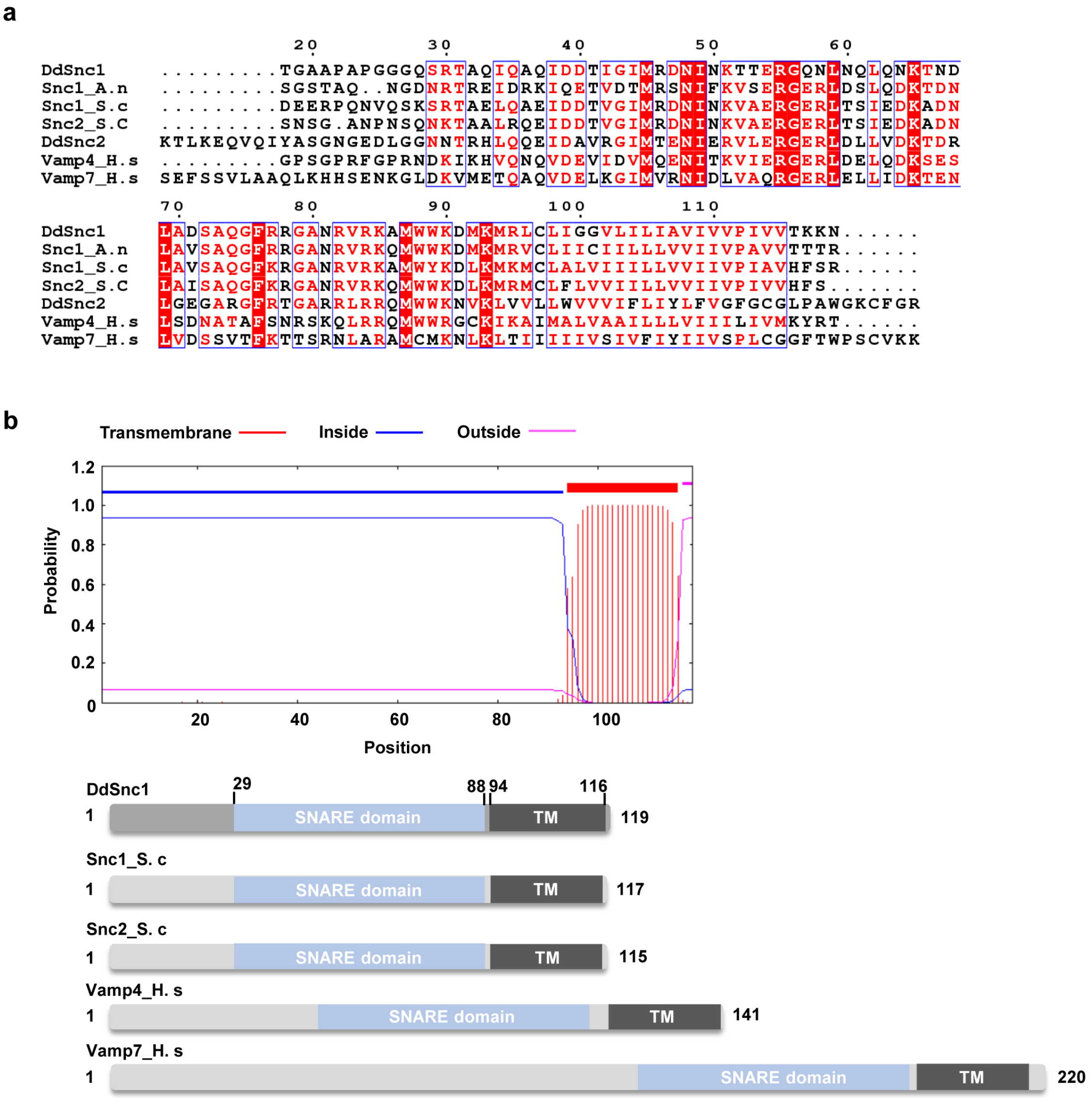

Supplementary Fig. 6. Sequence alignments of selected Snc proteins and their predicted domain structure. a The Snc proteins encoded by *D. dactyloide*, *S. cerevisiae* (NP_009372.1, https://www.ncbi.nlm.nih.gov/protein/NP_009372.1 for Snc1, and NP_014972.3, https://www.ncbi.nlm.nih.gov/protein/NP_014972.3 for Snc2), *A. nidulans* (XP_682038, https://www.ncbi.nlm.nih.gov/protein/XP_682038), *Homo sapiens* (NP_001172056.1, https://www.ncbi.nlm.nih.gov/protein/NP_001172056.1 for Vamp4, and NP_001138621.1, https://www.ncbi.nlm.nih.gov/protein/NP_001138621.1 for Vamp7) were aligned. Conserved amino acids are noted in blue boxes. Similar amino acids are indicated by red text, and identical amino acids are noted using the red background. The amino acid sequences of DdSnc1 and DdSnc2 are provided in Source Data file. **b** Predicted

functional domains of the *D. dactyloide* Snc1 protein and its homologs. The position of the transmembrane (TM) domain of DdSnc1 was predicted by TMHMM (Upper panel). The SNARE and transmembrane domains are noted using blue and black boxes, respectively (Bottom panel).

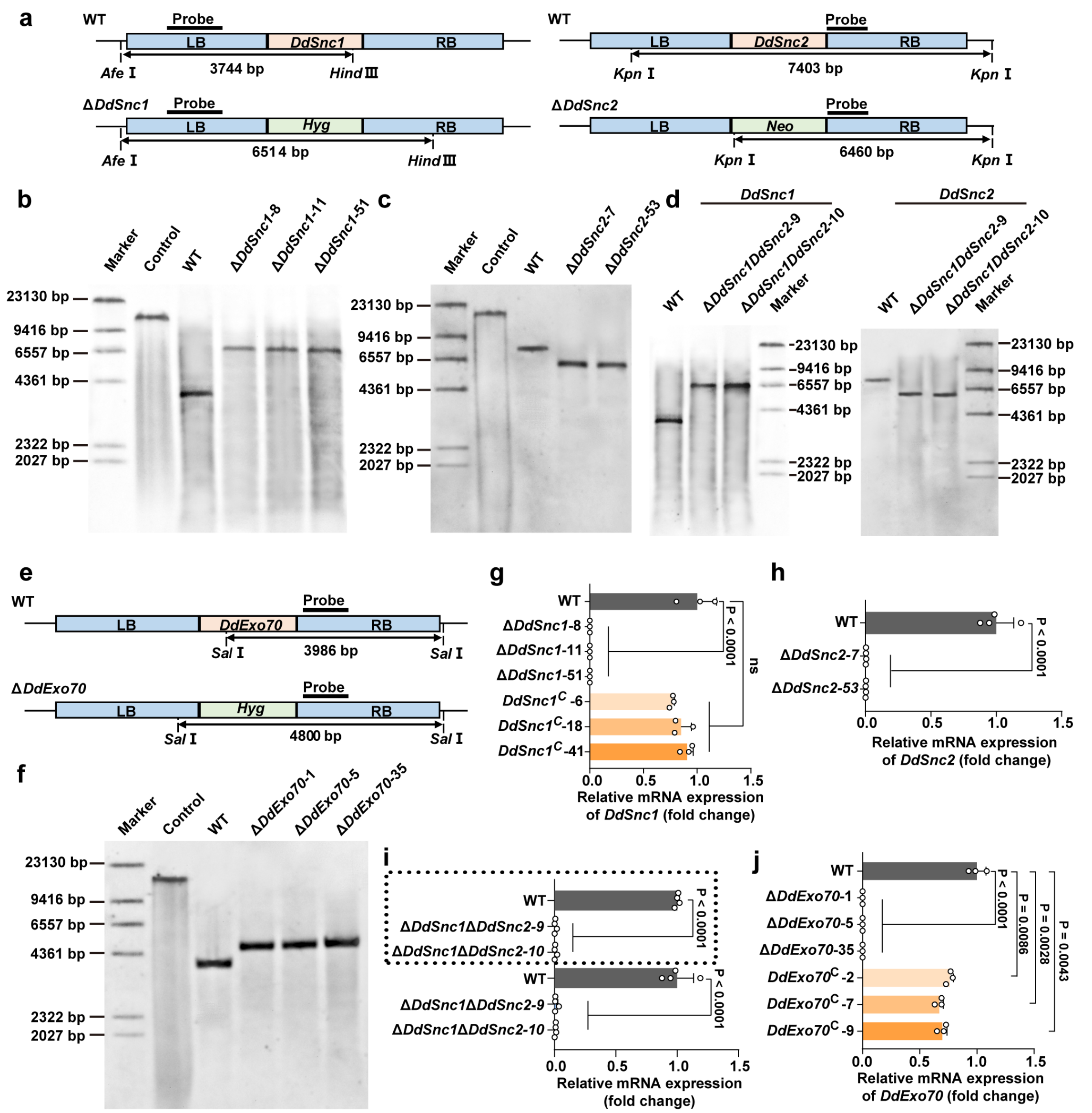

Supplementary Fig. 7. Gene disruption strategy and mutation verification by Southern analysis and RT-PCR. a Scheme of the deletion strategy for *DdSnc1* and *DdSnc2*. The genes were deleted via homologous recombination by flanking the hygromycin resistance cassette (*Hyg*) or geneticin resistance cassette (*Neo*) with ~3 kb upstream (LB) and downstream (RB) regions of *DdSnc1* or *DdSnc2*. **b** Confirmation of the *DdSnc1* deletion via Southern analysis. Genomic DNA was digested with *Afe*I and *Hin*dIII, and the location of probe and expected fragment length were indicated in **a**. The knockout plasmid containing the probe sequence served as a control (12122 bp). **c** Confirmation of the Δ*DdSnc2* deletion via Southern analysis. Genomic DNA was digested with *Kpn*I, and the location of probe and expected fragment length were indicated in **a**. The knockout

plasmid containing the probe sequence served as a control (12083 bp). **d** Confirmation of the deletion of both *DdSnc1* and *DdSnc2* in Δ*DdSnc1*Δ*DdSnc2* via Southern analysis. The expected fragment length is indicated in (**a**). **e** Scheme of the deletion strategy for *DdExo70*. **f** Confirmation of the Δ*DdExo70* deletion using Southern analysis. Genomic DNA was digested with *Sal*I, and the location of probe and expected fragment length were indicated in **e**. The knockout plasmid containing the probe sequence served as a control (12046 bp). Two times the experiment was repeated with similar results. **g, h** Transcripts from *DdSnc1* (**g,** n = 3 independent experiments) and *DdSnc2* (**h,** n = 4 independent experiments) in WT and mutant strains were measured using RT-PCR. Two-tailed t-test, mean ± SD. ns, not significant. **i** Transcripts of *DdSnc1* (dotted box) and *DdSnc2* in WT and Δ*DdSnc1*Δ*DdSnc2* were quantified (n = 4 independent experiments). Two-tailed t-test, mean ± SD. **j** Transcripts of *DdExo70* in WT and mutant strains were quantified (n = 3 independent experiments). Two-tailed t-test, mean ± SD. Source data are provided as a Source Data file.

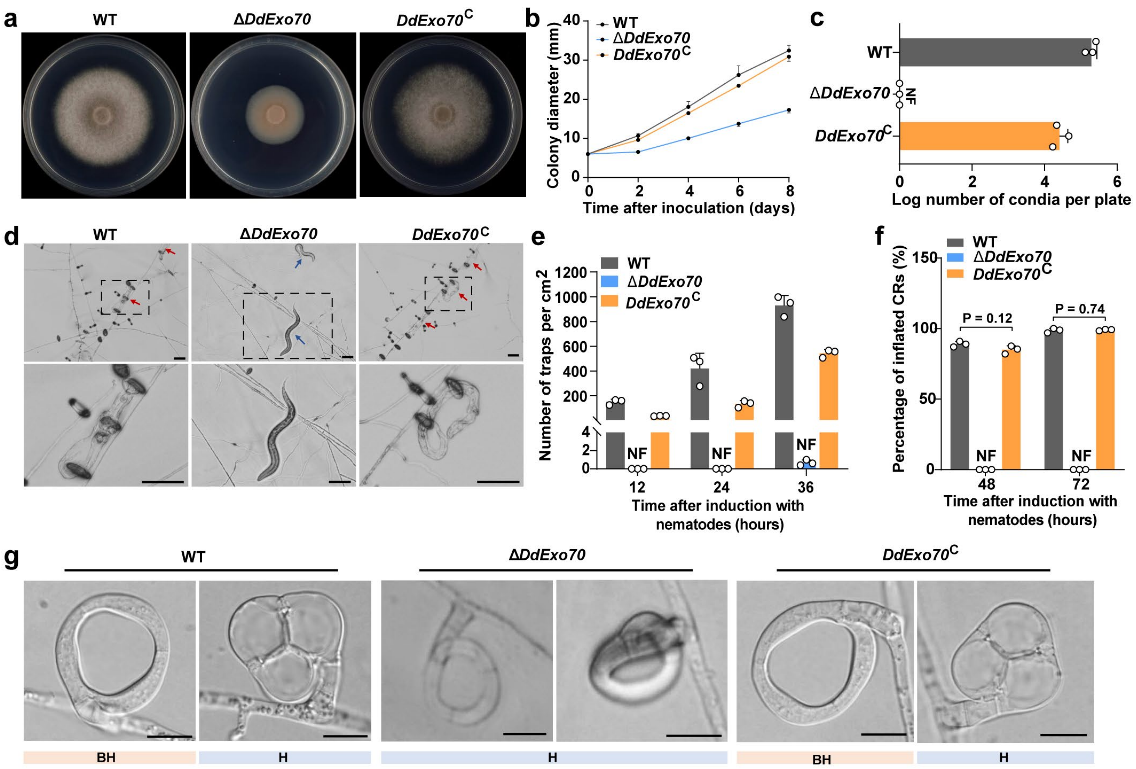

Supplementary Fig. 8. Requirement of DdExo70 for growth, conidiation, CR formation, and ring cell inflation. a Colonies of WT, Δ*DdExo70*, and *DdExo70*^{C} after culturing for 8 days on PDA. **b, c** The growth rate (**b**) and conidiation (**c**) of WT, Δ*DdExo70,* and *DdExo70*^{C}. The date shown are the means ± SD of three biological replicates. NF, not found. **d** Light micrographs showing CR formation and inflation 36 hours after introducing nematodes. Red and blue arrows denote captured and free nematodes, respectively. The lower panel shows magnified views of the dotted boxes in the upper panel. Scale bars = 50 µm. **e** Bar chart showing the numbers of CRs formed per cm^{2} 12, 24, and 36 hours after introducing nematodes. The date shown are the means ± SD of three biological replicates. NF, not found. **f** Bar chart showing the percentages of inflated CRs by heat stimulation applied 48 and 72 hours after introducing nematodes. The date shown are the means ± SD of three biological replicate. Two-tailed t-test. NF, not found. **g** Morphological characteristics of CRs before (BH) and after (H) stimulation with water at 55°C in WT, Δ*DdExo70* and *DdExo70*^{C}. Scale bars = 10 µm. Source data are provided as a Source Data file. Data shown is a representative from three independent experiments.

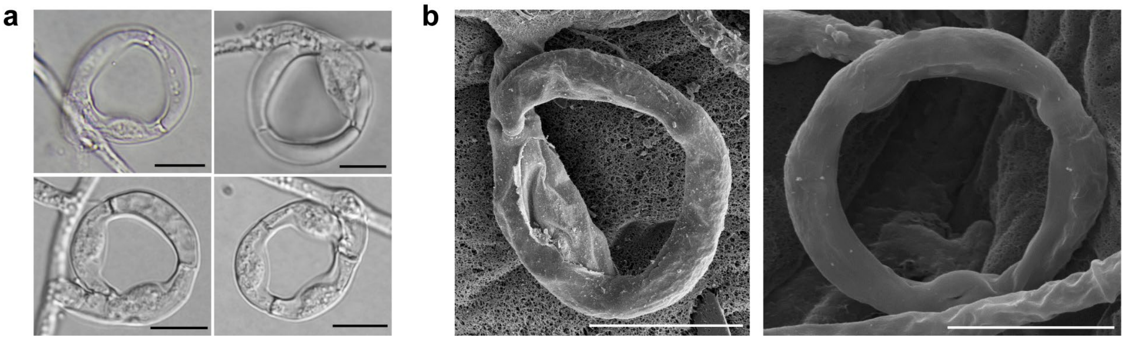

Supplementary Fig. 9. Partially inflated CRs of Δ*DdSnc1*. Light microscopic images (**a**) and scanning electron micrographs (**b**) of partially inflated CRs. Scale bars = 10 μm. Representative images from two independent experiments are shown.

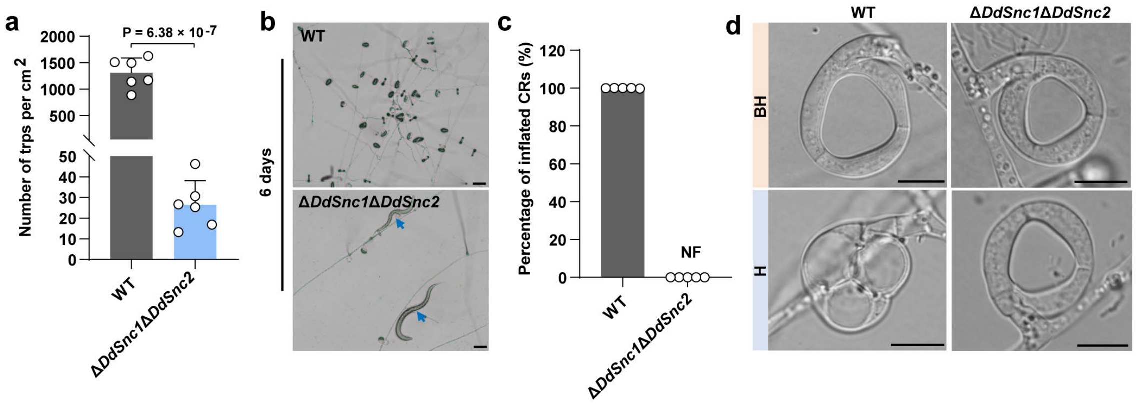

Supplementary Fig. 10. Phenotypic characteristics of Δ*DdSnc1*Δ*DdSnc2*. a Bar chart showing the numbers of CRs formed per cm^2 6 days after introducing nematodes (n = 5 biological replicates). Two-tailed t-test, mean ± SD. **b** Light micrographs showing CR formation 6 days after introducing nematodes. Blue arrows denote free nematodes. Scale bars = 50 µm. **c** Bar chart showing the percentages of inflated CRs by stimulating with water at 55ºC 6 days after introducing nematodes (n = 5 microscopic fields of view from 2 biological replicates). Mean ± SD. NF, not found. **d** Morphological characteristics of CRs before (BH) and after (H) stimulation with 55ºC water in WT and Δ*DdSnc1*Δ*DdSnc2*. Scale bars = 10 µm. Source data are provided as a Source Data file. Data shown is a representative from three independent experiments.

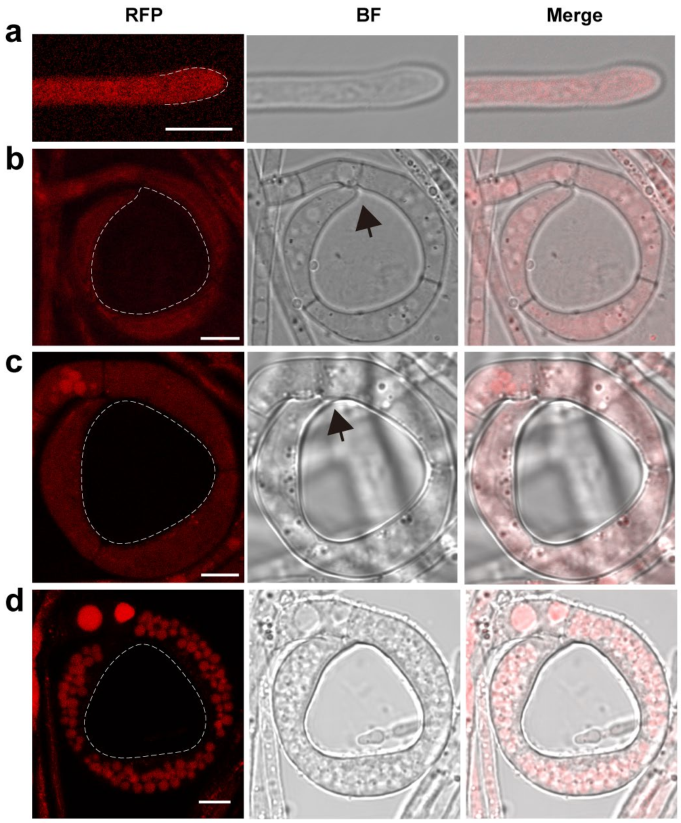

Supplementary Fig. 11. RFP signal during CR morphogenesis. RFP signals in (**a**) hyphal tips, (**b**) incompletely formed CR with incomplete fusion points (noted by black arrows), (**c**) immature CR with complete fusion points (noted by black arrows), and (**d**) mature CR are shown. Scale bars = 6 μm. Representative images from three independent experiments are shown.

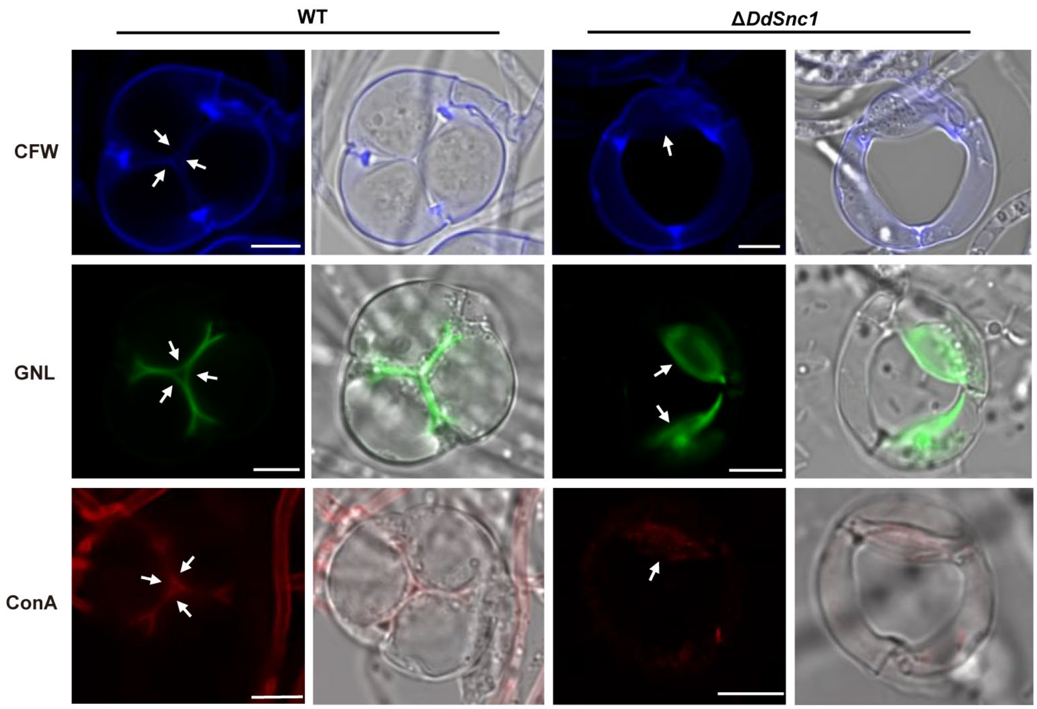

Supplementary Fig. 12. Cell wall constituents of the WT and Δ*DdSnc1* ring cells. Inflated CRs of WT and partially inflated CRs of Δ*DdSnc1* were stained with CFW, GNL, and ConA to label chitin, mannose residues, and α-glucopyranosyl residues, respectively. White arrows denote the inner cell wall. Scale bars = 6 μm. Representative images from three independent experiments are shown.

Supplementary Table 1. List of the reagents used in this study

Reagent	Source	Identifier
Potato dextrose agar (PDA)	BDTM	Cat# BD-213400
Corn meal agar (CMA)	BDTM	Cat# BD-211132
Water agar (WA)	Solarbio	Cat# LA8010
FM4-64 dye	Invitrogen	Cat# T13320
Calcofluor White (CFW)	Sigma-Aldrich	Cat# 18909
Galanthus nivalis (GNL)	Vector Laboratories	Cat# FL-1241-2
Concanavalin A (ConA)	Vector Laboratories	Cat# RL-1002-25
4′,6-diamidino-2-phenylindole (DAPI)	Solarbio	Cat# C0065
Neutral red	Solarbio	Cat# G1310
Brefeldin A (BFA)	Sigma-Aldrich	Cat# B5936
Horse serum	HyClone	Cat# SH30074.03
Geneticin (G418)	Solarbio	Cat# G8160
Hygromycin B	Solarbio	Cat# H8080
Trizol	Invitrogen	Cat# 15596026
All-in-One First-Strand cDNA Synthesis SuperMix	TransGen	Cat# AT341
FastStart Universal SYBR Green Master (ROX)	Roche	Cat# 04913914001

Supplementary Table 2. List of the primers used in this study

**Resistance gene**	
HYG-F	ttgcaagacctgcctgaaaccgaactgccc
HYG-R	aaccaagctctgatagagttggtcaagacc
NEO-F	ggctatgactgggcacaaca
NEO-R	gataccgtaaagcacgaggaa
**Deletion and complementation of *DdSnc1***	
Snc1-5F	gtaacgccagggttttcccagtcacgaCGATTGGTTAGCAAGTGATATGGGC
Snc1-5R	cgaattcactggccgtcgttttacaacgAGCTGAAGGCGTGAGAAGATTCA
Snc1-3F	cgaaaatcattcctactaagatgggtatacCGAGTCGAACGATTGGAACGAT
Snc1-3R	cggagcattcactaggcaaccatggttactattCAGGTCAGGTATTCGTCCGATGT
Snc1-OF	ATGTCCGACCAGCCCTACGACC
Snc1-OR	ACGACTGCAGGAAGATATGTGAGCAGC
Snc1-UP	ACTGTGCGAGCCAGCTCATGATC
Snc1-DN	TGCTGCCTGATACCCGTAACGGAG
Snc1-CF	agatctagaggatcccccgactagtGAGAATGGAGTCTATGCGCATCGATTG
Snc1-CR	atttaaatccgtttaaacggcgcgccTGTCCAGATAATCGGCCGTGAGCAC
**Deletion and complementation of *DdExo70***	
Exo70-5F	gtaacgccagggttttcccagtcacgaCTGTCCATTGCGGATGAGAGTGA
Exo70-5R	cgaattcactggccgtcgttttacaacgGTCGTCGGAGGTGGAGTGTGTAA
Exo70-3F	cgaaaatcattcctactaagatgggtatacCCGTCCGATTGCATCTGTAGTTAT
Exo70-3R	cggagcattcactaggcaaccatggttactattGGTGATTACCAGTTTCACGACCT
Exo70-OF	AGTCGGTCCAGCCAATCCTCAAAC
Exo70-OR	TCAACTGGCGGATGGTGTTCGAG
Exo70-OF2	AGTCGGTCCAGCCAATCCTCAAAC
Exo70-OR2	TACTGATTGCAGCTCGCGAGCACG
Exo70-UP	AGGTACTCATCCACGGCGACGTC
Exo70-DN	TGCGACAAGCCGATGAACACTTCC
Exo70-CF	agatctagaggatcccccgactagtACGGCAGTGTAGATTGATAGCGGCTG
Exo70-CR	atttaaatccgtttaaacggcgcgccTGTTGGATTGGCTGATCCACAGCC
**Deletion of *DdSnc2***	
Snc2-5F	gtaacgccagggttttcccagtcacgaAGCTGAGTTAGTGGAGGGTCCTC
Snc2-5R	cgaattcactggccgtcgttttacaacgATTGAGGGGCTGGTGTGATCTGG
Snc2-3F	cgaaaatcattcctactaagatgggtatacACACGGCATCAAGTTGTCTCGG
Snc2-3R	cggagcattcactaggcaaccatggttactattATCCTGCAGCTCAAGCTCGTG

Snc2-OF	TCGCTCATAATACGACCGTCCTC
Snc2-OR	TGTTCGCCGTACGTGTACGTGAG
Snc2-UP	TAAGTCTCTCGCTAGCCTCAATGCC
Snc2-DN	ATATGTTCTCGCAGCTTCTCGGTG
**RT-PCR**	
Tublin-RT-F	ATGGCACATCCGATCTCCAGC
Tublin-RT-R	AAGAGCTGGCCGAAGGGACCA
Snc1-RT-F	ATGTCCGACCAGCCCTACGACC
Snc1-RT-R	TGTCCCTCATGATGCCAATGGTGTC
Snc2-RT-F	TCGCTCATAATACGACCGTCCTC
Snc2-RT-R	TGTTCGCCGTACGTGTACGTGAG
Exo70-RT-F	AGTCGGTCCAGCCAATCCTCAAAC
Exo70-RT-R	TGCGGATGGTGCTCTCCTCATGC
**Fluorescent label of DdSnc1**	
Snc1-R-UP	agatctagaggatcccccgactagtGAGAATGGAGTCTATGCGCATCGATTG
Snc1-R-DN	atttaaatccgtttaaacggcgcgccTGTCCAGATAATCGGCCGTGAGCAC
RFP-UP	TCTTCCTGCAGTCGTGACAAAGAAGAATatggtgagcaagggcgaggagg
RFP-DN	GGTGGATGCGGTATAATTCCTTGGAATCActtgtacagctcgtccatgcc
**Fluorescent label of DdSnc2**	
Snc2-G-UP	TTCAAACACCGCCAATACCGCCATCATGCCGACTCCCCTCCTTTAG
Snc2-G-DN	AACAGCTCCTCGCCCTTGCGCATGCGACCGAAACATTTGCCCCA
GFP-UP	GCTTGGGGCAAATGTTTCGGTCGCATGCGCAAGGGCGAGGAGCTG
GFP-DN	GCATAGAGAAGATCACCTATTGGTAATTCACTTGTAGAGCTCGTCCAT

Uncropped blots for Supplementary Fig. 7

Supplementary Fig. 7d **Supplementary Fig. 7b**

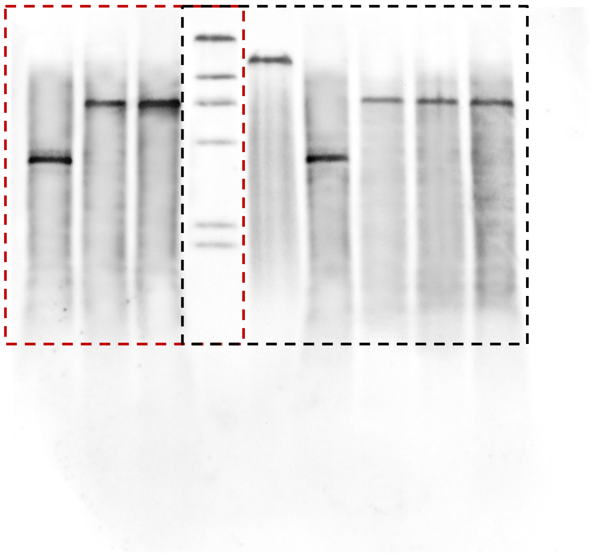

Supplementary Fig. 7d **Supplementary Fig. 7c**

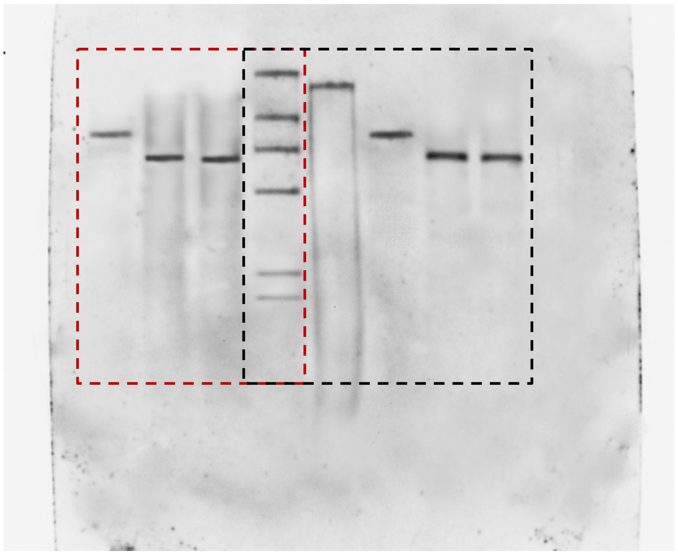

Supplementary Fig. 7f

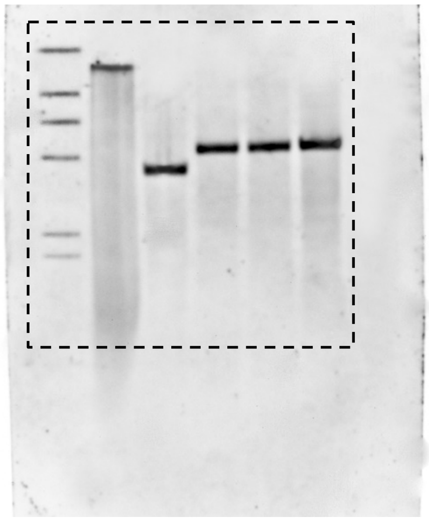

Supplement: Supplementary file 1 — Supplementary Information [file 41467_2023_43235_MOESM1_ESM.pdf]
