# Supplementary material for: A palisade-shaped membrane reservoir is required for rapid ring cell inflation in Drechslerella dactyloides
Source: Nat Commun. 2023 Nov 15;14:7376. doi: 10.1038/s41467-023-43235-w (PMC10651832; doi:10.1038/s41467-023-43235-w)
Supplement: Supplementary file 3 — Description of Additional Supplementary Files [file 41467_2023_43235_MOESM3_ESM.pdf]

Description of Additional Supplementary Files

File Name: Supplementary Movie 1
Description: A nematode getting caught by a CR, related to Fig. 1m.

File Name: Supplementary Movie 2
Description: Development of a CR, related to Fig. 1a.

File Name: Supplementary Movie 3
Description: Rapid ring cell inflation, related to Fig. 1f.

File Name: Supplementary Movie 4
Description: A nematode entering and escaping from an immature CR, related to Fig. 1l.

File Name: Supplementary Movie 5
Description: A nematode getting caught by a CR formed by wild type, related to Fig. 4j.

File Name: Supplementary Movie 6
Description: A nematode entering and escaping from a CR formed by *ΔDdSnc1*, related to Fig. 4k.

File Name: Supplementary Movie 7
Description: A nematode caught by a CR formed by *DdSnc1*^{C}, related to Fig. 4l.
