# Supplementary material for: A palisade-shaped membrane reservoir is required for rapid ring cell inflation in Drechslerella dactyloides
Source: Nat Commun. 2023 Nov 15;14:7376. doi: 10.1038/s41467-023-43235-w (PMC10651832; doi:10.1038/s41467-023-43235-w)
Supplement: Supplementary file 11 — Reporting Summary [file 41467_2023_43235_MOESM11_ESM.pdf]

nature portfolio

Corresponding author(s): Xingzhong Liu, Meichun Xiang, Yani Fan

Last updated by author(s): Oct 23, 2023

Reporting Summary

Nature Portfolio wishes to improve the reproducibility of the work that we publish. This form provides structure for consistency and transparency in reporting. For further information on Nature Portfolio policies, see our Editorial Policies and the Editorial Policy Checklist.

Statistics

For all statistical analyses, confirm that the following items are present in the figure legend, table legend, main text, or Methods section.

n/a	Confirmed	
☐	☒	The exact sample size (*n*) for each experimental group/condition, given as a discrete number and unit of measurement
☐	☒	A statement on whether measurements were taken from distinct samples or whether the same sample was measured repeatedly
☐	☒	The statistical test(s) used AND whether they are one- or two-sided<br>*Only common tests should be described solely by name; describe more complex techniques in the Methods section.*
☒	☐	A description of all covariates tested
☒	☐	A description of any assumptions or corrections, such as tests of normality and adjustment for multiple comparisons
☐	☒	A full description of the statistical parameters including central tendency (e.g. means) or other basic estimates (e.g. regression coefficient) AND variation (e.g. standard deviation) or associated estimates of uncertainty (e.g. confidence intervals)
☐	☒	For null hypothesis testing, the test statistic (e.g. *F*, *t*, *r*) with confidence intervals, effect sizes, degrees of freedom and *P* value noted<br>*Give P values as exact values whenever suitable.*
☒	☐	For Bayesian analysis, information on the choice of priors and Markov chain Monte Carlo settings
☒	☐	For hierarchical and complex designs, identification of the appropriate level for tests and full reporting of outcomes
☒	☐	Estimates of effect sizes (e.g. Cohen's *d*, Pearson's *r*), indicating how they were calculated

Our web collection on statistics for biologists contains articles on many of the points above.

Software and code

Policy information about availability of computer code

Data collection	Microscopy images were acquired using Nikon/Eclipse T2 microscope; Fluorescence images were acquired and analyzed using a TCSSP5 confocal microscope; Ultrastructural images were obtained using a Hitachi HT7700 transmission electron microscope, while scanning electron micrographs were generated using a QUANTA 200 scanning electron microscope; qRT-PCR was performed on Bio-Rad/CFX Connect Real-Time PCR system.
Data analysis	Figure preparing: Adobe illustrator CC 2019;<br>For plotting data, preparing graphs, and statistical analysis: GraphPAD Prism, version 8;<br>All constructs and primers design for gene editing: SnapGene, version 4.3.6;<br>Microscopy Image analysis: Fiji (ImageJ), version 2.0.0-rd-43;<br>Video editing: Wondershare Filmora, version 9.1.4.12;<br>Orthologues identification: BLASTP searches (http://blast.ncbi.nlm.nih.gov/Blast.cgi)<br>Conserved functional domain predictions: PFAM (http://pfam.xfam.org/), InterProScan (http://www.ebi.ac.uk/interpro/) ;<br>Putative transmembrane domains predictions: TMHMM (https://services.healthtech.dtu.dk/)<br>Sequence alignments: Clustalomega (https://www.ebi.ac.uk/Tools /msa/clustalo/)

For manuscripts utilizing custom algorithms or software that are central to the research but not yet described in published literature, software must be made available to editors and reviewers. We strongly encourage code deposition in a community repository (e.g. GitHub). See the Nature Portfolio guidelines for submitting code & software for further information.

Data

Policy information about availability of data

All manuscripts must include a data availability statement. This statement should provide the following information, where applicable:

- Accession codes, unique identifiers, or web links for publicly available datasets
- A description of any restrictions on data availability
- For clinical datasets or third party data, please ensure that the statement adheres to our policy

All data are available in the main text or the supplementary materials. The whole-genome shotgun sequence of strain 29 is available in GenBank (accession # JAGTWJ000000000; BioSample SAMN18837316; BioProject # PRJNA723920) (http://www.ncbi.nlm.nih.gov/bioproject/723920). The amino acid sequences of DdSnc1, DdSnc2, and DdExo70, in addition to the cDNA sequences of DdSnc1, DdSnc2, and DdExo70, have been made accessible in the Source Data file. All other relevant data supporting the findings of this study are available on request. Source data are provided with this paper.

Research involving human participants, their data, or biological material

Policy information about studies with human participants or human data. See also policy information about sex, gender (identity/presentation), and sexual orientation and race, ethnicity and racism.

Reporting on sex and gender	Not Applicable to this study
Reporting on race, ethnicity, or other socially relevant groupings	Not Applicable to this study
Population characteristics	Not Applicable to this study
Recruitment	Not Applicable to this study
Ethics oversight	Not Applicable to this study

Note that full information on the approval of the study protocol must also be provided in the manuscript.

Field-specific reporting

Please select the one below that is the best fit for your research. If you are not sure, read the appropriate sections before making your selection.

☒ Life sciences ☐ Behavioural & social sciences ☐ Ecological, evolutionary & environmental sciences

For a reference copy of the document with all sections, see nature.com/documents/nr-reporting-summary-flat.pdf

Life sciences study design

All studies must disclose on these points even when the disclosure is negative.

Sample size	The determination of the sample size was grounded in the principles of ensuring ample data for robust statistical power and dependable assessments. In the context of measuring the lengths of fused vesicles within constricting rings or evaluating the diameters of constricting rings, our approach involved the analysis of 5 to 10 constricting rings within each experimental group. Within each individual ring, we performed length measurements of the constituent strings at 3 randomly selected locations. For the mutant characterization and the assessment of inhibitor treatments, a minimum of 3 biological replicates was employed to enhance the reliability and accuracy of our findings. Furthermore, to investigate the inflation rate of these constricting rings, we observed 10 microscopic fields of view from 2 biological replicates.
Data exclusions	None
Replication	All experiments were repeated ≥ 3 times. Similar results were obtained from two independently conducted experiments.
Randomization	Nematodes were introduced randomly into the plates across all experimental groups to stimulate the formation of constricting rings. To examine the inflation rate of the constricting rings, fields of view were selected randomly on the plates under the microscope.
Blinding	Our study does not involve clinical trials and human participants, blinding was not relevant to this study.

Reporting for specific materials, systems and methods

We require information from authors about some types of materials, experimental systems and methods used in many studies. Here, indicate whether each material, system or method listed is relevant to your study. If you are not sure if a list item applies to your research, read the appropriate section before selecting a response.

Materials & experimental systems

n/a	Involved in the study
☒	☐ Antibodies
☒	☐ Eukaryotic cell lines
☒	☐ Palaeontology and archaeology
☐	☒ Animals and other organisms
☒	☐ Clinical data
☒	☐ Dual use research of concern
☒	☐ Plants

Methods

n/a	Involved in the study
☒	☐ ChIP-seq
☒	☐ Flow cytometry
☒	☐ MRI-based neuroimaging

Animals and other research organisms

Policy information about studies involving animals; ARRIVE guidelines recommended for reporting animal research, and Sex and Gender in Research

Laboratory animals	Caenorhabditis elegans
Wild animals	No wild animals were used in this study.
Reporting on sex	We used hermaphrodite worms for our reported analyses. We did not perform sex-based analysis because C. elegans does not naturally produce true females.
Field-collected samples	No filed-collected samples were used in this study.
Ethics oversight	No ethical guidance is required for work with C. elegans.

Note that full information on the approval of the study protocol must also be provided in the manuscript.
